# Differential Contributions of Dorso-Ventral and Rostro-Caudal Prefrontal White Matter Tracts to Cognitive Control in Healthy Older Adults

**DOI:** 10.1371/journal.pone.0081410

**Published:** 2013-12-02

**Authors:** Maren Strenziok, Pamela M. Greenwood, Sophia A. Santa Cruz, James C. Thompson, Raja Parasuraman

**Affiliations:** Department of Psychology, George Mason University, Fairfax, Virginia, United States of America; French National Centre for Scientific Research, France

## Abstract

Prefrontal cortex mediates cognitive control by means of circuitry organized along dorso-ventral and rostro-caudal axes. Along the dorso-ventral axis, ventrolateral PFC controls semantic information, whereas dorsolateral PFC encodes task rules. Along the rostro-caudal axis, anterior prefrontal cortex encodes complex rules and relationships between stimuli, whereas posterior prefrontal cortex encodes simple relationships between stimuli and behavior. Evidence of these gradients of prefrontal cortex organization has been well documented in fMRI studies, but their functional correlates have not been examined with regard to integrity of underlying white matter tracts. We hypothesized that (a) the integrity of specific white matter tracts is related to cognitive functioning in a manner consistent with the dorso-ventral and rostro-caudal organization of the prefrontal cortex, and (b) this would be particularly evident in healthy older adults. We assessed three cognitive processes that recruit the prefrontal cortex and can distinguish white matter tracts along the dorso-ventral and rostro-caudal dimensions –episodic memory, working memory, and reasoning. Correlations between cognition and fractional anisotropy as well as fiber tractography revealed: (a) Episodic memory was related to ventral prefrontal cortex-thalamo-hippocampal fiber integrity; (b) Working memory was related to integrity of corpus callosum body fibers subserving dorsolateral prefrontal cortex; and (c) Reasoning was related to integrity of corpus callosum body fibers subserving rostral and caudal dorsolateral prefrontal cortex. These findings confirm the ventrolateral prefrontal cortex's role in semantic control and the dorsolateral prefrontal cortex's role in rule-based processing, in accordance with the dorso-ventral prefrontal cortex gradient. Reasoning-related rostral and caudal superior frontal white matter may facilitate different levels of task rule complexity. This study is the first to demonstrate dorso-ventral and rostro-caudal prefrontal cortex processing gradients in white matter integrity.

## Introduction

Why do healthy older people decline in the efficiency of cognitive control, a cognitive function strongly dependent on PFC that includes working memory (WM), episodic memory, and reasoning [1], [2]? Older people present something of a puzzle for understanding the relation between brain change and cognitive change. They show a pattern of reduced PFC-dependent cognitive control in tandem with increased PFC activation [3]. Because changes in white matter integrity appear to be the most important neural correlate of cognitive aging [4], specific white matter tracts likely have a role in age-related modulation of the way PFC-dependent functions are integrated within and outside of PFC.

We describe a novel approach for understanding white matter integrity in older adults by advancing the hypothesis that the integrity of specific white matter tracts is related to cognitive functioning in a manner consistent with the organization of the PFC. We test specific predictions about how PFC organization is reflected in the integrity of projecting white matter tracts. Associations between white matter integrity and cognitive functions in older individuals can be informative not only about the neurocognitive organization of the PFC but also about possible age-related changes in that organization.

Cognitive control relies to a large degree on lateral prefrontal lobe grey matter function [5]–[7], which appears to be organized according to dorso-ventral and rostro-caudal processing gradients. Along the dorso-ventral axis, dorsolateral PFC (dlPFC) encodes task rules that enable the transformation of perception into action, whereas ventrolateral PFC (vlPFC) exerts control over semantic knowledge [8]–[10]. Superimposed on this dorso-ventral organization is a rostro-caudal organization that is based on processing complexity: anterior prefrontal regions represent complex rules and relationships between stimuli, whereas posterior PFC structures represent simple relationships between stimuli and behavior [9], [11]–[14].

Cognitive control relies not only on grey matter but also on white matter tracts subserving cortico-cortical and cortico-subcortical integration [15]–[17]. However, theoretical accounts of the functionality of dorso-ventral and rostro-caudal differentiations of cognitive processes are based mainly on functional imaging studies and have not been tested in the PFC's white matter architecture. Given that age-related white matter changes appear to be a significant neural substrate of cognitive aging [4], [18], there are important reasons to assess the role of PFC white matter organization in older adults.

First, fMRI-based accounts of these organizational principles provide little information about the *integration* of PFC-dependent cognitive functions within PFC and extra-prefrontal systems. Examining the links between structural neural organization and cognition will deepen understanding about how the PFC generates and integrates information in the service of cognitive control at a systems level. Information about increases or decreases in integrity of the white matter tracts that connect brain regions may, for example, help to interpret task-related brain activation increases or decreases that are commonly found in older compared to younger adults.

Second, it is important to know whether dorso-ventrally and rostro-caudally organized cognitive control processes are associated with specific inter- and intra-hemispheric structural connectivity patterns reflecting different mechanisms of PFC-dependent cognitive control. For example, there is evidence of a role in WM for intra-hemispheric dorso-dorsal connections between the frontal and parietal lobes, viz., dorsal superior longitudinal fasciculus (SLF II) [19], [20]. This suggests the SLF II is involved in top-down control of PFC during parietal cortex-dependent maintenance processes in WM [21]. There is also evidence that anterior corpus callosum is involved in inter-hemispheric inhibitory or excitatory processes in PFC-dependent WM control [22]–[26]. For vlPFC-dependent episodic memory, on the other hand, evidence suggests reliance on intra-hemispheric, but not inter-hemispheric connections [23], [27].

Third, studying correlations between PFC organization and white matter integrity can reveal whether the specific tracts important for cognitive control vary with age. The aged brain experiences decreased white matter integrity particularly in the PFC [28]–[30], which is bilaterally connected through fibers of the anterior corpus callosum. Reduced integrity in the anterior corpus callosum has been associated with decline in PFC-dependent cognitive control in older adults [23], [26], [31], whereas increased PFC white matter integrity has been observed following cognitive control training in older adults [24]. The evidence that older adults activate the PFC bilaterally in contrast to the unilateral pattern in young people [32], [33] suggests the anterior corpus callosum has a prominent role in PFC-dependent cognitive control in older adults. However, to date, the correspondence between portions of the anterior corpus callosum and specific PFC-dependent cognitive functions has not been studied. We asked whether associations between anterior callosal white matter integrity and cognitive control in older adults subserve dorso-ventrally or rostro-caudally organized PFC grey matter.

### White matter integrity as a reflection of PFC-dependent cognitive control

Although PFC white matter integrity has been associated with cognitive function in both young and older adults, theoretical accounts of dorso-ventral and rostro-caudal organizational principles of the PFC have not been previously tested in white matter. Regarding episodic memory, correlations with white matter integrity revealed reliance on fronto-thalamic connections via the anterior internal capsule and temporal lobe white matter [23], [27]. Regarding WM, associations have been found with the SLF II in young people [19], [20] and with the genu of the corpus callosum across the adult age range [22]–[24], [26]. Regarding reasoning, associations have been observed between PFC white matter and frontal, parietal, and temporal tracts [22], [34].

Reductions in white matter integrity appear to be an important substrate of cognitive aging but no previous study has related white matter integrity to the organization of the PFC. Accordingly, in the present study we examined, in a population of healthy older adults, three cognitive processes that recruit the PFC and can distinguish white matter tracts along the dorso-ventral and rostro-caudal dimensions - episodic memory, WM, and reasoning. We hypothesized that the integrity of specific white matter tracts is related to cognitive functioning in a manner consistent with the postulated dorso-ventral and rostro-caudal organization of the PFC, as described in the following paragraphs.

### Dorso-ventral organization of the lateral PFC

Both imaging and lesion studies reveal a clear dorso-ventral processing organization in the dlPFC (Brodmann area, BAs 8, 9, 46, 6 rostral) that can be observed in WM, episodic memory, and reasoning tasks. The dlPFC appears to be involved in encoding task rules aimed at transforming perception into action [9], [35]. For example, it is activated when words are organized according to a mathematical pattern [36], or reordered according to a physical property of the object that the word represents [37], and during analytical reasoning [38]. This processing is facilitated by dorsal neocortical pathways and connections between dlPFC and adjacent prefrontal and premotor regions [39]. The vlPFC (BAs 44, 45, 47) appears to be involved in the encoding and selection of items held in semantic memory [8], [40]–[43]. It is activated during the encoding of remembered words and pictures [41], semantic analysis and phonological rehearsal of source memory for nouns [44], and selection and inhibition of familiar letters [45]–[47]. This processing is facilitated by connections between vlPFC and the inferior and superior temporal cortices [10].

This evidence is consistent with the view that the dlPFC codes task-relevant rules to transform a wide range of sensory inputs into actions (“how” function) [9], [48], thereby accounting for a range of dlPFC-dependent rule-based functions such as re-organizing words and objects. The anatomical connectivity of the dlPFC with the dorsal parietal cortex (dorso-dorsal pathways), and vlPFC with temporal association areas (ventro-ventral pathways), lends support to the described functional specializations [10], [39], [49]–[51]. What has not been investigated previously are the specific white matter tracts that are critical for specific aspects of dorsally- and ventrally-mediated cognitive control.

### Rostro-caudal organization of the lateral PFC

Current views of the rostro-caudal organization within the lateral PFC are also based on functional imaging and lesion evidence. Anterior PFC is thought to facilitate processing of domain-integrative, relationally complex, abstract, and temporally extended information, whereas posterior PFC facilitates processing of domain-specific, less complex, concrete, and temporally close information [10], [12], [52]–[54]. The rostro-caudal gradients in the vlPFC evolved in the context of a cytoarchitecture that likewise varies regionally in complexity. The vlPFC consists of distinct subdivisions that gradually increase in granularity from caudal to rostral regions [55]–[57]. Also, anatomical connectivity in some dlPFC regions suggests distinct rostral and caudal pathways: the more rostrally located BA 9 and BA 46 are connected with the multimodal superior temporal cortex and rostral superior temporal gyrus, whereas the more caudally located BA 8 is connected with visuospatial parietal and posterior visual temporal cortices [10].

Fuster [13] argued that rostro-caudal dlPFC gradients reflect the ability of the lateral frontal cortex to exert cognitive control over temporally segregated components of behavior sequences [5], [53]. Specifically, premotor cortex encodes the stimulus at hand, caudal lateral PFC represents temporally close perceptual context, and rostro-lateral PFC encodes temporally distant stimulus context. In accordance with this idea, Koechlin et al. [53] showed that activation in lateral frontal regions of sensory, contextual, and episodic control increased in magnitude in a caudal-to-rostral manner, and exerted causal top-down influences among the regions. A series of related functional and lesion imaging studies using a hierarchical response selection task revealed a similar pattern of lateral rostro-caudal frontal organization [52], [58] that may signal conflict resolution in ambiguous stimuli by imposing additional processing steps in progressively rostral regions [59]. Less is known about possible rostro-caudal gradients of semantic control in the vlPFC, although one study found that solving anagrams of increasingly abstract words evoked brain activations in progressively anterior areas among ventro-, dorso-, and rostro-lateral PFC regions [60]. Evidence for the role of white matter integrity in rostro-caudal PFC organization in cognitive processing would advance the current knowledge about the organization of the PFC.

### Predictions for PFC white matter organization in the current study

We assessed three cognitive functions that recruit the PFC and can be used to distinguish white matter tracts along the dorso-ventral and rostro-caudal dimensions. We asked whether associations between the regional integrity of white matter microstructure and specific cognitive function support the organizational principles within the PFC.

The dorso-ventral organization was assessed by comparing the integrity of tracts subserving episodic memory and WM. We predicted that episodic memory, assessed with the logical memory subtest of the Wechsler Memory Scale III (WMS III) [61], would rely on the integrity of anterior corona radiata tracts (fronto-thalamic connections), thereby supporting vlPFC-dependent semantic control. This prediction is based on (a) diffusion-tensor imaging (DTI)-based evidence of a relation in older adults between white matter integrity in these tracts and episodic memory performance [23], [27] and (b) fMRI evidence for vlPFC-dependent semantic control [8], [9]. Because the WMS logical memory test measures the retrieval aspect of semantic cognition - the executive process of goal-directed activation of semantic knowledge - it was chosen to assess ventral PFC-dependent semantic control [62]–[70]. We further predicted that WM, assessed with the Letter Number Sequencing (LNS) subtest of the Wechsler Adult Intelligence Scale III (WAIS III) [71], would be associated with integrity in anterior transcallosal connections between the bilateral dlPFC (corpus callosum genu/body), thereby facilitating dlPFC-dependent task rule encoding. This prediction is based on previous evidence that anterior callosal white matter integrity is related to WM in older adults [22], [23], [26], evidence of increased corpus callosum genu integrity following WM training in older adults [24], and fMRI evidence for dlPFC-dependent task rule encoding [9], [10]. Finally, we predicted that reasoning, assessed with the matrix reasoning subtest of the WAIS III, would be associated with integrity in anterior PFC white matter based on previous fMRI studies on reasoning function [38], [72].

The rostro-caudal organization was assessed by comparing WM, associated with relatively posterior PFC areas (dlPFC), and reasoning ability, associated with the anterior PFC. Specifically, we predicted that abstract thinking, assessed with the matrix reasoning subtest of the WAIS III [71], would show dependence on anterior PFC tracts that connect the frontal pole with parietal association cortices. We predicted that this would be particularly true for participants who solve WAIS III items with more complex designs compared to participants that solve simpler designs. Our prediction is based on findings from two previous studies in older adults that showed reliance of reasoning ability on PFC tracts [22], [34] and the reviewed fMRI evidence of the rostral PFC's representation of complex rules and relationships between stimuli [9], [11]–[14].

## Methods

### Ethics Statement

All procedures were approved by the George Mason University Institutional Review Board and written informed consent was obtained from each participant prior to testing. Participants received monetary compensation for their time and effort.

### Sample

Initially, 142 individuals with no self-reported history of neurologic and psychiatric disorders were considered for this study, which is part of a longitudinal study on healthy aging. Clinical signs for cognitive dysfunction were assessed with the Mini Mental State Exam (MMSE, cut-off: raw score<25) [73]. In addition, T2-weighted FLAIR MRI images sensitive for lesion detection were acquired and evaluated by a neuroradiologist to screen for significant injuries and brain pathology, such as tumors and stroke. Three adults were excluded based on their MMSE scores, but no one was excluded based on the neuroradiological evaluations. One adult had to be excluded because of a poor ventral prefrontal diffusion signal. In four adults, matrix reasoning and LNS scores were not available. Therefore, the final sample consisted of 134 healthy adults aged 41 to 86 (mean = 63.3, SD = 10.3). The study group was predominantly female (63.4%), right-handed (85.8%), and White (85.6%). The remainder of the sample was classified as Hispanic (5.5%), African-American (3.9%), or Other (5%).

### Neuropsychological assessment

Neuropsychological assessment was performed as part of a larger test battery in the Healthy Aging Study at the George Mason University Psychology Department in Fairfax, Virginia. The Mini Mental State Exam was used to screen for cognitive impairment [73]. Episodic memory was assessed with the logical memory subscale of the WMS III [61]. Participants were instructed to retain as much information as possible from an aurally presented short biographical story. Episodic memory measures the quantity of semantic information retrieved from the story which is captured by the number of correctly recollected semantic units (raw score). WM was assessed with the LNS subscale of the WAIS III [71]. Participants were read sequences of alternating digits and letters in scrambled order and required to immediately retrieve them in ascending numeric followed by alphabetical order. LNS measures the efficiency with which individuals manipulate sequential order. A raw score was computed based on the number of accurately completed trials. The matrix reasoning test of the WAIS III [71] was used to assess abstract reasoning at a range of stimulus complexity. Participants were shown incomplete designs that started with simple comparisons of single dimensions (e.g., shape or color) and then gradually increased in complexity in the relations between stimuli dimensions (e.g., color, shape, and orientation had to be considered simultaneously). They were then asked to select a design part to complete each design from a choice of five parts. The number of correctly completed designs (raw score) was recorded. In order to test our hypothesis of dorso-ventral and rostro-caudal PFC white matter organization of cognitive control, we excluded any assessments that were not from standardized tests and any that did not assess PFC-mediated cognitive control.

### MRI data acquisition

MRI images were collected with a Siemens Allegra head-only MRI scanner and a standard quadrature transmit-receive head coil. Axial images were acquired using a diffusion-weighted single-shot EPI sequence with 12 gradient directions (1000 s/mm2; TE = 75 ms; TR = 10000 ms; slice thickness = 3 mm, 50 slices, acquisition matrix = 128 mm×128 mm) and 3 repetitions.

### Diffusion imaging data processing

MRI data processing was performed using the FDT toolbox (version 2.0) implemented in FSL 4.1.8. [74]. Preprocessing of diffusion-weighted images included correction for head movements and eddy currents, creating a brain mask on non-diffusion-weighted reference images, and voxel-wise fitting of the diffusion tensor model yielding fractional anisotropy (FA) maps for each participant. FA is a frequently used index of white matter integrity that quantifies the magnitude and directionality of water diffusion in the brain [75], [76]. It is assumed that white matter has an oriented fiber structure which results in faster movement of water molecules along the lengths of axons and slower movement in perpendicular directions due to molecular barriers. FA values can range from zero to one, where zero indicates non-directional (isotropic) and one indicates perfectly directional (anisotropic) diffusion. The cellular basis of anisotropic water diffusion in the brain is not fully understood to date. Research indicates that it is not exclusively related to the ordered arrangement of white matter fibers but also depends on the density of axon packing, axonal diameter, axon volume fraction, and degree of myelination [76]–[78].

Tract-based spatial statistics (TBSS) [79] was then applied to compute whole-brain correlations between FA and cognitive performance. The TBSS processing stream included removal of brain edge outliers from the diffusion tensor fitting, non-linear registration of individual FA maps to the FMRIB58_FA standard space, and linear transformation of the registration parameters into 1 mm isotropic MNI152_T1 space. Normalized FA maps were averaged to create a mean FA map across subjects. Then, the mean FA map was thresholded at 0.2 to define a white matter skeleton mask – a common set of voxels across subjects that is used for further analyses [79]. Finally, all subjects' aligned FA data were projected onto the mean FA skeleton that was fed into a voxel-wise covariance analysis.

### Statistical analyses of fractional anisotropy

A one-sample voxel-wise whole brain t-test (general linear regression, GLM) was computed on the skeletonized mean FA maps of all participants (N = 134). In the GLM, the raw scores from the cognitive assessments of matrix reasoning, LNS, and episodic memory (immediate recall) were included as regressors of interest, while age, sex, handedness, z-transformed MMSE scores, and the z-transformed number of years of education served as nuisance regressors.

In addition, the study group was divided by the level of participants' performance on the matrix reasoning score using the split-half method to assess whether performance on complex versus simple items of this measure is related to rostro-caudal PFC organization. This analysis was first carried out on the whole brain FA maps. To reduce the number of comparisons in these smaller groups of participants (N = 67 in each group), we additionally performed statistical analyses using a TBSS-derived mean white matter skeleton mask specifically created to include only PFC white matter voxels. Whole brain and PFC-only voxel-wise t-tests were performed separately on the high and low-performing participants using matrix reasoning, age, sex, handedness, z-transformed MMSE scores, and the z-transformed number of years of education as predictors.

For all analyses, positive and negative cognition-FA associations were examined. FSL's Randomise tool with threshold-free cluster-enhancement (tfce) thresholding and family-wise error (FWE) correction for multiple comparisons was used to obtain correlational maps. [80]. Images were overlaid onto a rendered brain volume (MNI152_T1_1 mm) for display purposes. The JHU White-Matter Tractography Atlas and Juelich Histological Atlas were used to determine the identity and probabilities of white matter tracts that revealed significant correlations between FA and cognition. Adjacent grey matter structures were identified using Talairach labels, also implemented in FSL 4.1.8 [74].

### Probabilistic tractography analyses

Single-seed probabilistic tractography implemented in FSL's FDT toolbox was used to create white matter path distributions from white matter areas that showed significant correlations with episodic memory, WM, and reasoning (TBSS analysis results). This analysis has the advantage that likely cortical targets can be estimated which is not possible with TBSS. First, local diffusion directions for the primary and secondary fiber orientations in each voxel were computed with a fully automated Bayesian estimation method (BedpostX) [81]. Then, diffusion image registration parameters were computed for transformation to MNI152_T1_1mm space for each individual. Tractography was performed on each individual's data using Probtrackx to reconstruct fiber tracts that pass through the seeded white matter areas. Specifically, 5000 samples were drawn through the probability distributions of each seed voxel, probabilistic tracking propagated at a step length of 0.5 mm, and a curvature threshold of 0.2 was applied to limit the analysis to likely fiber pathways that turn approximately 80 degrees or less [81].

**Figure 1:**
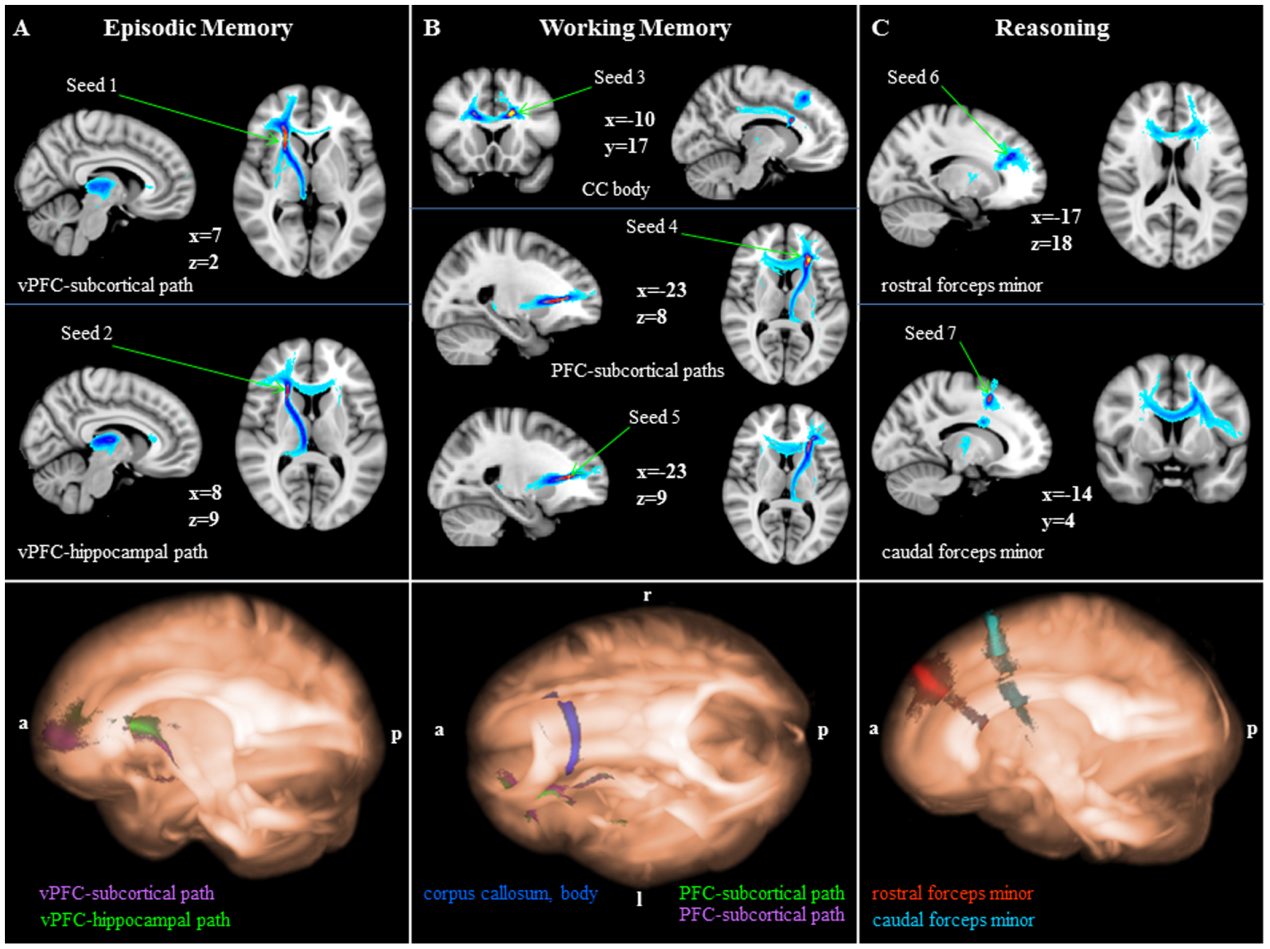
Probabilistic tractography results. Top row: Pathways for episodic memory (A), working memory (B), and reasoning (C) are shown in 2D overlaid onto MNI152_T1_1 mm standard space (radiological convention). The color code represents the number of participants for which connectivity distributions were found in a given voxel with warm colors indicating larger and cold colors indicating smaller participant overlap (yellow  =  largest overlap, light blue  =  smallest overlap). Seed locations are indicated by arrows. Bottom row: Pathways for episodic memory (A), working memory (B), and reasoning (C) are shown in 3D using MRIcroGL overlaid onto MNI152_T1_1 mm standard space (vPFC, ventral prefrontal cortex; CC, corpus callosum; a, anterior; p, posterior; l, left, r, right).

**Figure 2:**
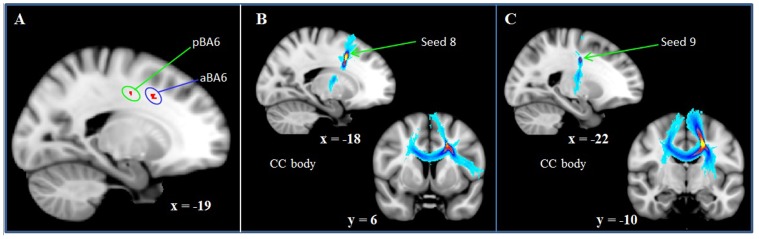
PFC correlations between FA and less complex reasoning. TBSS (A) and probabilistic tractography (B, C) results are displayed for reasoning in low-performing individuals. A: Reasoning in low-performing participants relies on white matter subjacent to the left anterior (aBA6) and left posterior (pBA6) BA6 (PFC-only analysis; p = 0.08, corrected). B, C: Pathways tracked from aBA6 (B) and pBA6 (C) are shown overlaid onto MNI152_T1_1 mm standard space (radiological convention). The color code represents the number of participants for which connectivity distributions were found in a given voxel with warm colors indicating larger and cold colors indicating smaller participant overlap (yellow  =  largest overlap, light blue  =  smallest overlap). Seed locations are indicated by arrows (CC, corpus callosum).

Small 2 mm spherical seed masks (Figs. 1; 2B,C) were created in 1 mm isotropic MNI152_T1 standard space for nine locations. Seven of these locations were based on the whole-brain FA-cognition correlation results from the three positive statistical TBSS contrasts carried out in the entire sample; two more locations were based on the analysis in the subgroup of participants that solved less complex matrix reasoning items (described in Results, FA-cognition correlations derived from the TBSS analysis). The correlation maps for episodic memory, WM, and matrix reasoning in the subsample of participants that solved less complex items yielded highly localized peak intensities. We therefore used voxels that yielded the highest probability of belonging to a certain tract and the greatest signal intensity as the centers for the seeds that were based on these correlations. To determine the voxels with the highest probability of belonging to a certain tract, the JHU Juelich White-Matter Tractography Atlas was used first and, in cases where this atlas did not yield a tract label, the Juelich Histological Atlas was used instead to classify the peak voxels in each seed region and obtain their probability values. The statistical map that resulted from the whole-brain FA-matrix reasoning correlational analysis in the entire sample did not reveal localized peaks. Therefore, rostral and caudal white matter voxels were selected from significant white matter voxels subjacent to the dlPFC. The rostro-caudal cut-off was set to y = 34 (MNI 1 mm space) based on results from a previous fMRI study that assessed rostro-caudal dlPFC gradients [53]. Voxels with the greatest signal intensities from the rostral and caudal white matter areas subjacent to dlPFC grey matter served as centers for the seeds derived from this correlation analysis.

Based on these criteria, the following seeds were used for the fiber tractography analyses: right inferior fronto-occipital fasciculus (IFOF; seed 1: MNI, 23, 23, 3; episodic memory); right anterior thalamic radiation (seed 2: 23, 19, 11; episodic memory); left corpus callosum body (seed 3: −16, 17, 28, WM); left anterior thalamic radiation (seed 4: −25, 31, 10; WM, from left IFOF/anterior thalamic radiation cluster); IFOF (seed 5: −25, 31, 7; WM, from left IFOF/anterior thalamic radiation cluster); left rostral dlPFC (seed 6: −11, 45, 36; matrix reasoning, entire sample); left caudal dlPFC (seed 7: −11, 12, 57; matrix reasoning, entire sample); anterior BA6 (seed 8: −19, 9, 39; matrix reasoning, low performers); posterior BA6 (seed 9: −18, −10, 41; matrix reasoning, low performers).

For each seed region, the raw connectivity map of each participant was computed and thresholded at 50% to include pathways that were evident in at least half of the samples that were drawn through each voxel of each of the seed regions. The thresholded maps were then binarized and summed for all participants to create seven group maps that yielded the highest values in areas with the most overlap of pathways among participants for the seven seed regions.

## Results

### Whole-brain FA-cognition correlations (entire sample)

Whole-brain correlations between FA and each of the three cognitive measures were carried out on episodic memory, WM, and reasoning in the entire sample. We found the following positive FA-cognitive control correlations (negative contrasts did not yield significant results).

#### Episodic memory

**Figure 3:**
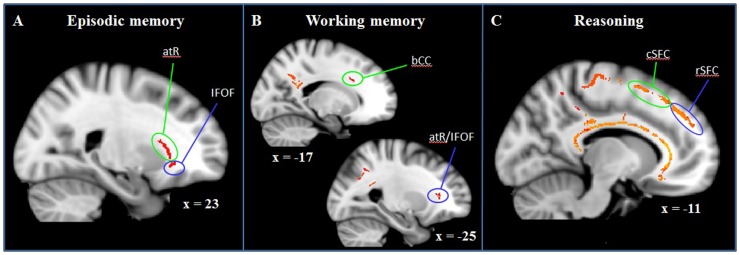
Whole-brain correlations between FA and cognitive measures (entire sample). Color-coded white matter tracts represent significant positive correlations between FA and episodic memory (A; p<0.05, corrected), working memory (B; p = 0.07, corrected), and reasoning (C; p<0.05, corrected). Episodic memory relies on right anterior thalamic radiation (atR) and right inferior fronto-occipital fasciculus (IFOF); Working memory is facilitated by left body of corpus callosum fibers (bCC) and left atR/IFOF fibers; Reasoning depends on integrity in caudal (cSFC) and rostral (rSFC) superior frontal cortex, premotor cortex, corpus callosum (all bilateral, but displayed exemplary for the left hemisphere), and additional frontal, parietal, temporal, and subcortical white matter (not shown here). Results are overlaid onto MNI152_T1_1 mm standard space.

Higher FA in the right vlPFC, including the IFOF (peak probability, 55%, JHU White-Matter Tractography Atlas) and anterior thalamic radiation (37%, JHU White-Matter Tractogaphy Atlas), was associated with better performance on the WMS logical memory (immediate recall) test (p<0.05; FWE corrected, based on the threshold-free cluster-enhanced statistical image), indicating that ventro-ventral long-range association fibers and fibers that convey information from subcortical thalamic nuclei to the PFC facilitate episodic memory (Fig. 3A).

#### Working memory

Higher FA in two PFC clusters were associated with better LNS performance: in the left body of the corpus callosum (98%, Juelich Histological Atlas) and in the left IFOF/anterior thalamic radiation (45%/32%, JHU White-Matter Tractogaphy Atlas; Fig. 3B). These results approached significance (p = 0.07, FWE corrected, based on the threshold-free cluster-enhanced statistical image). Peak signal intensities were found in the left body of the corpus callosum near the anterior intraparietal cortex (IPC; 96%, Juelich Histological Atlas, p<0.05; tfce corrected). These findings indicate that frontal and parietal white matter tracts are implicated in WM.

#### Reasoning

In the PFC, we found greater FA in bilateral fibers along the entire anterior to posterior extent of the superior frontal cortex in close proximity to individual superior frontal gyri associated with better reasoning performance (p<0.05; FWE corrected, based on the threshold-free cluster-enhanced statistical image; Fig. 3C). These fibers were not classified as any of the major long-range association or fronto-subcortical fibers by the white matter atlases implemented in FSL. Also in the PFC, there were significant correlations between reasoning and FA in the bilateral genu and body of the corpus callosum. This widespread prefrontal white matter network extended into the premotor cortex, superior and inferior parietal cortices, left posterior parahippocampal gyrus, bilateral anterior corona radiata and bilateral splenium of the corpus callosum.

### FA-cognition correlations associated with reasoning complexity

To test our hypothesis concerning reasoning and PFC tracts connecting the frontal pole with parietal association cortices, whole-brain and PFC-only correlations between FA and matrix reasoning were carried out in two subsamples: high (matrix reasoning raw score>17) and low (matrix reasoning raw score<18) performing individuals. On the whole brain level, no significant associations were found between FA and matrix reasoning. Justified by our hypothesis, analyses were also restricted to PFC white matter tracts. These revealed a trend for a positive correlation between FA subjacent to two areas in the left superior frontal gyrus (near anterior and posterior BA 6; p = 0.08, FWE corrected, based on the threshold-free cluster-enhanced statistical image; Fig. 2A) and matrix reasoning performance in the individuals who performed poorly on the matrix reasoning test. No significant correlations or trends were found in the group of high performing individuals. Negative contrasts did not yield significant results.

### Probabilistic tractography results

Seed masks were used to track pathways associated with the three cognitive measures and revealed distinct connectivity patterns in ventral, dorso-rostral, and dorso-caudal PFC white matter.

#### Episodic memory

Tracking from the first seed region that we identified as the right IFOF based on the TBSS analysis revealed that these fibers are primarily right anterior thalamic radiation fibers passing through the anterior limb of the internal capsule to connect the ventral PFC with the thalamus (Fig. 1A, seed 1). At the level of the right putamen, some fibers from the seeded area continue their course into the putamen. We therefore refer to these tracts as ventral PFC-subcortical paths to account for their cortico-subcortical course. These tracts subserve medial (BA 10) and lateral (BA 45) ventral PFC regions to facilitate prefrontal-thalamic and prefrontal-putamen communication. A second fiber orientation in the first seed is evident in this map that indicates inter-hemispheric vlPFC connections via the corpus callosum genu.

Relatively dorsal to these ventral PFC-subcortical connections run pathways that we identified as the anterior thalamic radiation based on the TBSS results (Fig. 1A, seed 2). Tractography revealed that their medial and lateral ventral PFC targets are similar in location to those found with the first seed mask (BAs 10, 45). However, their extra-frontal terminations take a more posterior course into the right hippocampus. We therefore refer to these connections as a ventral PFC-hippocampal path. A second fiber orientation is evident that passes through the corpus callosum genu thereby facilitating inter-hemispheric communication between the bilateral ventral PFC.

#### Working memory

Tracking from the third seed region revealed corpus callosum body fibers (Fig. 1B, seed 3) that interconnect the bilateral superior frontal gyri (BA 6). A second fiber pathway connects the dorsal PFC with the posterior cingulate cortex (BA 31). Tracking from the fourth and fifth seed region (identified as the left anterior thalamic radiation based on the TBSS analysis) revealed PFC-subcortical pathways passing through the anterior limb of the internal capsule, with cortical targets in the vlPFC (BA 45) and dlPFC (BA 9; Fig. 1B, seed 4). Some of these fibers continue beyond the level of the thalamus to end in the left hippocampus, thereby indicating a PFC-hippocampal pathway. A second fiber orientation connects the left PFC with its contralateral counterpart (BA 9). Fiber tracking from the fifth seed, located immediately ventral to the anterior thalamic radiation location and initially identified as the left IFOF, revealed pathways that were very similar to those revealed by tracking from the fourth seed (Fig. 1B, seed 5).

#### Reasoning (entire sample)

The sixth seed region was created to assess white matter tracts subjacent to the left rostral superior frontal gyrus. Tracking from that seed revealed forceps minor fibers that are extensions of the corpus callosum body providing inter-hemispheric connections with the right rostral superior frontal gyrus (Fig. 1C, seed 6). Other fibers that pass through the sixth seed have subcortical targets that are less certain but may include the thalamus, putamen, and/or brain stem. Similarly, the seventh seed mask was used to assess tracts subjacent to the left caudal superior frontal cortex. The main fiber orientation revealed by that seed were forceps minor fibers that are extensions of the corpus callosum body connecting the caudal superior frontal gyrus with its contralateral counterpart. A second fiber orientation revealed in this map appears to run perpendicular to the corpus callosum and connects the left caudal superior frontal region with the left inferior frontal gyrus (Fig. 1C, seed 7). Before reaching the inferior frontal gyrus, some of these fibers take a medial course via the posterior limb of the internal capsule ending in the thalamus.

#### Reasoning (low performing individuals)

The eighth and ninth seed regions were created to assess white matter tracts subjacent to the left caudal superior frontal gyrus (anterior and posterior BA 6). Tracking from the eighth seed revealed corpus callosum body fibers that interconnect the bilateral anterior BA 6 (Fig. 2B). A second fiber orientation revealed tracts to the left inferior frontal gyrus. Some additional fibers were found that connect the thalamus with BA6. Tracking from the ninth seed region revealed corpus callosum body fibers that interconnect the bilateral posterior BA 6 (Fig. 2C). A second fiber orientation was found revealing connections between BA 6 and the thalamus.

## Discussion

Consistent with our hypothesis that white matter integrity reflects organizational gradients in PFC previously seen in functional imaging studies, we found distinct intra- and inter-hemispheric PFC white matter fibers associated with PFC-mediated cognitive function.

### Regional variation in white matter integrity along the dorso-ventral PFC axis

Our findings in white matter support the dorso-ventral distinction claimed to exist in gray matter between semantic and rule-based processing [9]. Specifically, we identified (a) ventral prefrontal tracts that subserve communication in episodic memory and (b) dorsal prefrontal tracts that subserve communication in working memory.

Regarding ventral aspects of PFC organization, previous functional imaging studies found that episodic memory recruits the vlPFC [40]–[42], [44], thalamus [82], putamen [83], and hippocampus [8], [43], [84], [85]. Nevertheless, the full circuitry of episodic memory is unknown to date and our findings are informative about the ventral circuitry of episodic memory. We found that episodic memory relies on ventral cortico-subcortical circuitry (ventral prefrontal-thalamic, ventral prefrontal-putamen, and ventral prefrontal-thalamo-hippocampal pathways). This is in line with two previous DTI studies that revealed that anterior thalamic radiation fibers carry information between the vPFC and thalamus [23], [27]. Both lateral and medial vPFC are connected with the medial dorsal thalamus and putamen [86]–[89]. Even though the ventral prefrontal (vPFC) terminations that we identified here (BA 10, BA 45) are known to be critically involved in episodic memory retrieval [90], less is known about the role of fronto-thalamic and fronto-putamen interactions in higher-order cognition [15]–[17]. Some research indicates that fronto-thalamic loops have a general role in modulating response magnitude, firing mode, and synchrony of cortical neurons in the service of cognition [91]. Our results confirm the importance of fronto-thalamic interactions.

Our results extend what was previously known about the circuitry subserving episodic memory by finding a ventral prefrontal-thalamo-hippocampal pathway not previously reported. Previous animal research revealed the existence of multiple hippocampal-thalamic connections that interact with the PFC and other brain structures in the service of recollection memory [92]. With the spatial resolution of the current tractography data, we cannot determine the thalamic nuclei through which these tracts pass. One possibility is that these fibers belong to an anterior thalamic feed-forward system thought to convey integrated hippocampal-thalamic signals to the PFC [92]. Such a system is well known from lesion work showing the importance of the hippocampus in using information derived from past experience to carry out a range of tasks. This pathway appears to be critical for short-term episodic memory – both hippocampus/PFC-mediated memory formation and PFC-mediated subsequent selection of memory [93]. The present findings confirm the importance of this pathway in human episodic memory.

Regarding the dorsal aspect of PFC organization, we predicted WM (measured using the LNS task) would rely on integrity of frontal and parietal white matter tracts based on previous functional [9], [10] and diffusion imaging evidence [19], [22]–[24], [26]. Specifically, we predicted an effect on WM performance of integrity in (a) corpus callosum body fibers that integrate information between the bilateral dlPFCs, and (b) fibers that connect the dlPFC with the parietal cortex (via the SLF II). We found a trend for WM reliance on corpus callosum body connections between the bilateral dlPFC, underscoring the important role of frontal inter-hemispheric integration in the service of WM. Our findings are also in accord with longstanding neuropsychological evidence from acallosal and commissurotomized patients and a growing body of diffusion-based research suggesting that corpus callosum fibers subserve the bilateral integration of cognitive functions [22], [23], [26], [94]–[96]. A large body of functional neuroimaging evidence suggests that the dlPFC mediates WM, including conditions requiring manipulation of information according to a rule [36], [97]–[101], as in the LNS task. Therefore, the corpus callosum body connections between the bilateral dlPFC appear to subserve rule-based information manipulation in WM.

We did not predict reliance of WM on white matter connections between the vlPFC and subcortical structures, but we did observe trend-level involvement of a left-sided vlPFC-thalamo-hippocampal pathway. That tract follows a path that is similar to the right-sided pathways that we found in our FA-episodic memory analyses. We speculate that the verbal nature of the LNS task requires communication between vlPFC and the thalamus/medial temporal lobe. The finding of WM reliance on vlPFC tracts does not align with the claimed dorso-ventral PFC distinction that is the focus of the current study.

### Regional variation in white matter integrity along the rostro-caudal axis

Our findings in the WM and reasoning domains support the existence of a rostro-caudal PFC white matter gradient previously claimed to exist in gray matter. That gradient reflects varying levels of processing complexity [9] that map to specific rostral and caudal frontal cortical areas [52], [53]. Comparing the current WM results with those from previous fMRI studies that investigated lateral PFC function differences along a rostro-caudal axis [52], [53], the dlPFC connections that we found appear to facilitate communication between the bilateral caudal dlPFC. This area corresponds approximately to the caudal dlPFC reported by Badre et al. [52] that was activated when participants had to simultaneously consider two stimulus characteristics. In the current study, participants had to simultaneously consider numerical and alphabetical orders to successfully complete the LNS task, which may explain the reliance of WM on caudal dlPFC white matter tracts found in the current study.

Regarding the rostral PFC organization, we predicted that associations with reasoning would be strongest in rostral PFC white matter based on functional evidence that anterior prefrontal regions are important in representations of complex rules and relationships between stimuli [12], [53], [97]. Correlations of FA with matrix reasoning that we observed revealed a wide-spread fronto-parietal-temporal-subcortical white matter network. This finding is consistent with evidence that integrity in several tracts subserves fronto-parieto-temporal and cortico-subcortical communication, shown to share common variance with reasoning ability [34]. Our findings are also in agreement with previous brain activation studies that showed fluid ability, deductive reasoning, and abstract thinking are dependent on dlPFC, vlPFC, and posterior parietal structures [38], [60], [72], [102].

A considerable portion of the tracts that we detected lay along a rostro-caudal axis in the dlPFC, extending into the more caudally located premotor cortex. Pathways tracked from rostral and caudal white matter underneath the dlPFC revealed the anterior corpus callosum subserving caudal and rostral PFC areas. The rostro-caudal gradient in the PFC is claimed to reflect increases in task rule complexity as the organizing principle of dlPFC rostro-caudal hierarchy [9]. We speculate that our observation that matrix reasoning performance relies on premotor, caudal dlPFC, and rostral dlPFC fibers is consistent with the increasing complexity of task rules implemented by the increasing number of stimuli dimensions (color, shape, orientation) that have to be considered as the matrix reasoning task progresses. However, these findings are not sufficiently specific to link complex reasoning to rostral and simple reasoning to caudal PFC white matter.

Another finding in the current study was particularly revealing about the role of white matter in rostral-caudal organization. We found a trend for FA-reasoning associations in the caudal dlPFC (left BA 6) in participants whose matrix reasoning ability was limited to solving easier items. Functional evidence of the PFC's rostro-caudal organization suggests that the caudal dlPFC, including BA 6, facilitates processing of domain-specific, less complex, concrete, and temporally close information, compared to the rostral PFC which is involved in the processing of complex, abstract, and temporally distant information [10], [12], [52], [53]. Our finding is in accordance with these previous accounts of rostro-caudal distinctions in PFC function as they suggest that performance with less complex reasoning demands is more strongly associated with connections between left BA 6 and both inferior frontal cortex and right BA 6, suggesting reliance of processing of simple rules and relationships on these pathways.

Our results do not support the notion of a rostro-caudal abstraction gradient in the ventrolateral PFC [9], [60] as we found both rostral (BA 10) *and* caudal (BA 45) ventral PFC regions involved in episodic memory. Previous research has shown that distinct rostral and caudal regions of the caudate nucleus are associated with the complexity of controlled processing of semantic information [103] and that multiple hippocampal-thalamic connections exist that interact with the PFC in the service of recollection memory [92]. Therefore, future research could be aimed at identifying rostro-caudal complexity gradients in subcortical regions, such as the thalamus, to further our understanding of prefrontal-subcortical organization in episodic memory.

### PFC organization and aging

In the current study, we sought to identify organizational principals of PFC function in white matter integrity in an aging population. One influential brain aging theory suggests that aging is associated with reductions in hemispheric asymmetries in brain function, and increased bilateral processing, claimed to be compensation for age-related decline in neurocognitive function [32], [33], [104]. The beneficial effects of bilateral processing are presumably facilitated through corpus callosum fibers in addition to concomitant bilateral intra-hemispheric engagement of white matter structure [105], [106]. We found evidence of bilateral processing via the corpus callosum for all three observed cognitive processes, suggesting that the integrity of the corpus callosum plays a role in the greater bilateral involvement of older brains on a range of tasks. Our findings of a distinct dorso-ventral and rostro-caudal PFC white matter organization confirm results from fMRI and lesion studies in younger populations. As such, our findings are important not only for showing that the greater bilateral involvement seen in gray matter in older people is also evident in white matter, but also for showing that nevertheless the organization of PFC-dependent cognitive control seen in the young is maintained in older adults.

### Summary and limitations

This is the first report of a relation between white matter integrity and PFC hierarchical organization. These findings complement a small functional imaging literature supporting current theories of PFC hierarchical organization. We obtained evidence for the existence of functional specializations in PFC white matter by showing (a) vlPFC-thalamo-hippocampal white matter subserves semantic control, (b) corpus callosum body fibers subserve task rule processing, and (c) anterior corpus callosum fibers connect bilateral rostral and caudal dlPFC fibers subserving reasoning. Thus, associations between the integrity of PFC white matter circuitry and cognitive performance confirm and extend evidence on PFC organization from brain activation studies. A few of our findings only approached significance and therefore have to be interpreted with caution. The current study is notable for its inclusion of a relatively large adult age range, reasonable sample size, and the control of several factors that have been shown to interact with white matter integrity and cognitive function, including age, sex, handedness, and education [30], [107]–[109]. It is one of only a few studies that have assessed white matter-cognition correlations on the whole-brain level therefore avoiding an a priori bias towards individual tracts. By using probabilistic tractography, we were able to show likely pathways that pass through the significant regions of FA-cognition correlation, thereby allowing the visualization of the terminal branches of tracts that are relevant in cognition.

TBSS is a sophisticated statistical method to assess correlations between diffusion-derived parameters of white matter integrity and cognitive performance for major white matter tracts. It allows simultaneous assessment of the integrity of important tracts without bias to a priori defined regions on well-aligned group data. Probabilistic tractography significantly strengthened the understanding of our TBSS findings and allowed us to perform a group overlay of fiber connections. On the downside, both TBSS and tractography suffer from a range of limitations, notably the applied tensor model that cannot satisfactory resolve the presence of two or more fiber orientations in a given voxel. In addition, partial volume averaging in single voxels that present with complex fiber relations such as crossing and “kissing” of fibers may result in inaccurate FA values [110]. Another limitation in the current study is that we could not directly compare our white matter findings to those from young adults.

## Conclusion

In sum, we found evidence for dorso-ventral and rostro-caudal distinctions that are embedded in a larger neocortical and subcortical scheme of dorso-dorsal and ventro-ventral connectivity. We speculate that cognition-FA associations result from a combination of experience-driven adaptation [111]–[113] and genetic predisposition [114]. Because age-related reductions in white-matter integrity appear to be the most important neural substrate of cognitive aging, particularly in the PFC [30], [115], [116], and white matter can be altered through cognitive training [24], [117], [118], associations between white matter integrity and cognitive functions in older individuals can lead to a greater understanding of the circuitry underlying the organization of the PFC and of brain health in a large proportion of people.
